# ORC6, Negatively Regulated by miR-1-3p, Promotes Proliferation, Migration, and Invasion of Hepatocellular Carcinoma Cells

**DOI:** 10.3389/fcell.2021.652292

**Published:** 2021-07-29

**Authors:** Hu Chen, Lequn Bao, Jianhua Hu, Dongde Wu, Xianli Tong

**Affiliations:** ^1^Department of Hepatobiliary and Pancreatic Surgery, Hubei Cancer Hospital, Tongji Medical College, Huazhong University of Science and Technology, Wuhan China; ^2^Department of Laboratory, Hubei Cancer Hospital, Tongji Medical College, Huazhong University of Science and Technology, Wuhan, China

**Keywords:** hepatocellular carcinoma, miR-1-3p, ORC6, microRNA, cells

## Abstract

**Background:**

In recent years, microRNA-1-3p (miR-1-3p) has been linked to the progression of multiple cancers, whereas little is known about its role in hepatocellular carcinoma (HCC). Herein, we investigated the function of miR-1-3p in HCC, and its regulatory function on origin recognition complex subunit 6 (ORC6).

**Methods:**

Quantitative real-time polymerase chain reaction (qRT-PCR) was performed for detecting the expression levels of miR-1-3p and ORC6 mRNA in HCC samples and cell lines. ORC6 expression at the protein level was quantified by Western blot. After gain-of-function and loss-of-function models were established, cell counting kit-8 (CCK-8) assays, Transwell assays, flow cytometry, and 5-Ethynyl-2′-deoxyuridine (EdU) assay were performed for examining cell proliferation, migration, invasion, cell cycle, and apoptosis. The targeting relationship between miR-1-3p and ORC6 was confirmed with bioinformatic analysis and dual-luciferase reporter assays.

**Results:**

The expression of miR-1-3p was reduced in HCC samples and cell lines. Overexpression of miR-1-3p suppressed the proliferation, migration, and invasion, and induced cell-cycle arrest and apoptosis of HCC cells, whereas the opposite effects were induced by miR-1-3p inhibition. ORC6 is identified as a novel target of miR-1-3p, the expression of which is negatively correlated with miR-1-3p expression in HCC tissues. ORC6 overexpression facilitated the proliferation, migration, invasion, and cell cycle progression, and reduced apoptosis of HCC cells, whereas the opposite effects were induced by ORC6 knockdown. What is more, ORC6 overexpression counteracted the biological functions of miR-1-3p in HCC cells.

**Conclusion:**

MiR-1-3p targets ORC6 to suppress the proliferation, migration, invasion, and cell cycle progression, and promote apoptosis of HCC cells.

## Introduction

Liver cancer, ranking as the fourth most common cancer worldwide, is a deadly disease. Hepatocellular carcinoma (HCC) is the main histological subtype of liver cancer (Villanueva, 2019). Currently, the advances in the treatment of HCC are limited, and the prognosis of HCC patients is still unsatisfactory, especially for those with metastatic disease or relapse (Khemlina et al., 2017; Greten et al., 2019; Marquardt et al., 2019; Raoul et al., 2019). It is necessary to explore the molecular mechanism of HCC progression to look for novel therapeutic targets to further improve the survival time of the patients.

More and more microRNAs (miRNAs) have been reported to participate in the tumorigenesis and progression of cancers. MiR-1-3p is identified as a tumor suppressor in various tumors. For instance, the expression of miR-1-3p is markedly reduced in bladder cancer tissues; by targeting BDNF, miR-1-3p constrains the proliferation and invasion and expedites the apoptosis of bladder cancer cells (Gao et al., 2018). Reportedly, miR-1-3p is downregulated in HCC cell lines such as Hep3B and HCCLM3, and miR-1-3p overexpression represses the proliferation of HCC cells (Zhang et al., 2019). Nonetheless, the role of miR-1-3p in HCC requires further clarification.

Origin recognition complex subunit 6 (ORC6) is crucial in DNA replication initiation (Balasov et al., 2009). In colorectal cancer tissues, ORC6 expression is markedly upregulated, associated with the invasion depth of the tumor and the survival time of the patients (Chesnokov et al., 2003). Additionally, ORC6 is associated with the tumorigenesis of prostate cancer (Wei et al., 2020). However, the roles of ORC6 in the development of HCC require further investigation.

In the present work, we report that the expression level of miR-1-3p is markedly downregulated in HCC tissues, whereas the expression level of ORC6 is upregulated. We also demonstrate that in HCC, miR-1-3p is a tumor suppressor, and ORC6 can facilitate cancer progression. What is more, we identify ORC6 as a novel target gene of miR-1-3p.

## Materials and Methods

### Clinical Samples

Forty patients with HCC who received hepatectomy in Hubei Cancer Hospital from May 2016 to May 2019 were enrolled in this study. HCC tissues and adjacent liver tissues were obtained. All the enrolled patients received no chemotherapy, radiation treatment, or target therapy before the surgery. Besides, blood samples from these HCC patients, along with healthy subjects receiving physical examination, were collected. All tissues and blood samples were preserved in liquid nitrogen. This work is supported by the Ethics Committee of Hubei Cancer Hospital, and written informed consents of the patients were obtained.

### Cell Culture and Transfection

HCC cell lines Hep3B, HepG2, HCCLM3, Huh7, and normal liver cells L02 were obtained from American Type Culture Collection (ATCC, Manassas, VA, United States). These cells were cultured in RPMI-1640 medium (Thermo Fisher Scientific, Waltham, MA, United States) supplemented with 10% fetal bovine serum (FBS) (Thermo Fisher Scientific, Waltham, MA, United States), 100 μg/ml streptomycin (Thermo Fisher Scientific, Waltham, MA, United States), and 100 U/ml penicillin (Thermo Fisher Scientific, Waltham, MA, United States) at 37°C in 5% CO_2_ in a humidified incubator.

MiR-1-3p mimics and its control (mimics control), miR-1-3p inhibitors and its control (inhibitors control), ORC6 overexpression plasmids (ORC6), empty vector (vector) siRNAs for ORC6 (si-ORC6-1 and si-ORC6-2), and their negative controls (si-NC) were obtained from GeneChem (Shanghai, China). Lipofectamine^TM^ 3,000 (Thermo Fisher Scientific, Waltham, MA, United States) was used for performing the transfection. Forty-eight hours after transfection, the cells were collected and quantitative real-time polymerase chain reaction (qRT-PCR) or Western blot was performed for examining the transfection efficiency.

### qRT-PCR

Total RNA from tissue samples and cell lines was extracted utilizing TRIzol reagent (Invitrogen, Carlsbad, CA, United States). Then, total RNA was utilized as the template for the synthesis of cDNA with PrimeScript^TM^ RT Reagent Kit (Takara, Shanghai, China). With cDNA as the template, qRT-PCR was performed on a 7500 Fast Real-Time PCR system (Thermo Fisher Scientific, Waltham, MA, United States), using a Bestar qPCR MasterMix kit (DBI Bioscience, Shanghai, China). U6 and GAPDH were used as the endogenous control. The quantification was performed with the 2^–ΔΔ*CT*^ method. The primers used in the study are listed in Table 1.

**TABLE 1:** Primers for qRT-PCR.

Gene name	Primer sequence
miR-1-3p	F: 5′-CAGTGCGTGTCGTGGAGT-3′
	R: 5′-GGCCTGGAATGTAAAGAAGT-3′
U6	F: 5′-CTCGCTTCGGCAGCACA-3′
	R: 5′-AACGCTTCACGAATTTGCGT-3′
ORC6	F: 5′- ACAAGGAGACATATCAGAGCTGT- 3′
	R: 5′- AGTGGCCTGGATAAGTCAAGAT- 3′
GAPDH	F: 5′-ACATCGCTCAGACACCATG-3′
	R: 5′-TGTAGTTGAGGTCAATGAAGGG-3′

### Cell Counting Kit-8 (CCK-8) Assay

After transfection, Huh7 and Hep3B cells were inoculated into 96-well plates with 2 × 10^3^ cells per well. The cells were cultured. CCK-8 solution (10 μl) (Dojindo Molecular Technologies, Kumamoto, Japan) was added to each well at 24, 48, 72, or 96 h. Then, the cells were further incubated for 2 h, and then the absorbance of the cells at 570 nm was measured.

### Transwell Assays

Transwell chambers (Millipore, Boston, MA, United States) were used for cell migration and invasion assays. In migration assays, cells in the upper compartment (2 × 10^4^ cells/well) were cultivated in serum-free RPMI-1640 medium, while the lower compartment was supplemented with RPMI-1640 medium containing 10% FBS. After 24 h of culture, migrated cells across the Transwell membrane were fixed with 4% paraformaldehyde, stained with 0.1% crystal violet solution, and counted in five randomly selected fields of view under an inverted microscope. In invasion assays, Matrigel-coated Transwell chambers were used, and the other procedures were the same.

### Analysis of Apoptosis

Huh7 and Hep3B cells were resuspended in 1 × binding buffer, and cell concentration was adjusted to 1 × 10^6^/ml. In each group, 100 μl of cell suspension was mixed with 5 μl of Annexin V/FITC staining solution, and 10 μl of 20 μg/ml propidium iodide (PI) staining solution (Beyotime, Shanghai, China), and incubated at room temperature for 15 min in the dark. Then, a flow cytometer (FACScan; BD Biosciences, San Jose, CA, United States) was used to examine the apoptosis of the cells.

### Analysis of Cell Cycle

Huh7 and Hep3B cells were harvested and rinsed with PBS. After the cells were fixed with 75% ethanol, the cells were stained with 100 μg/ml PI staining solution (Beyotime, Shanghai, China) at room temperature for 10 min. Then, a flow cytometer (FACScan; BD Biosciences, San Jose, CA, United States) was used to examine the cell cycle distribution of the cells.

### 5-Ethynyl-2′-Deoxyuridine (EdU) Assay

The proliferation of HCC cells was also performed using the Click-iT^®^ EdU Assay Kit (Invitrogen, Carlsbad, CA, United States). Huh7 and Hep3B cells in the logarithmic growth phase were harvested and trypsinized to prepare the single-cell suspension, and the cells were inoculated in a 96-well plate (5,000 cells/well). Then, 150 μl of 50 μmol/L EdU medium was added to each well, followed by the incubation for 2 h at 37°C. After washing the cells three times with PBS, 200 μl of PBS containing 4% paraformaldehyde was added to each well, with which the cells were fixed at room temperature for 40 min. Subsequently, 200 μl of glycine at a concentration of 2 mg/ml was added to each well and incubated with cells for 5 min on a decolorizing shaker. After discarding the solution, 300 μl of PBS was added to each to wash the cells again. After PBS was discarded, 200 μl of PBS containing 0.5% Triton X-100 was added to each well, and the cells were incubated for 30 min. After the cells were washed with PBS again, 200 μl of Apollo staining reaction solution was added to each well and incubated with cells for 30 min in the dark, and then 200 μl of DAPI solution was added to each well and incubated with cells for 30 min in the dark. Finally, the pictures of the cells were taken under a fluorescent inverted microscope, and the percentage of EdU positive cells was calculated.

### Western Blot

RIPA lysis was used to extract the total protein from the cells. Protein concentrations were quantified using a BCA Protein Assay Reagent kit (Thermo Fisher Scientific, Waltham, MA, United States). Subsequently, the protein extracts were separated utilizing SDS-PAGE and then transferred onto the PVDF membrane (Millipore, Boston, MA, United States). Then, the membranes were incubated with the primary antibody (anti-ORC6 antibody, 1:1,000, ab153993; anti-Bax antibody, 1:1,000, ab182733; anti-PCNA antibody, 1:1,000, ab92552; anti-MMP-2 antibody, 1:1,000, ab92536; anti-MMP-9 antibody, 1:1,000, ab76003, Abcam, Shanghai, China) at 4°C for 8 h. After the membranes were washed by TBST, the membranes were incubated with horseradish peroxidase-coupled secondary antibody (goat anti-rabbit IgG H&L, 1: 2,000, Beyotime, Shanghai, China) for 1 h at room temperature. Next, the membranes were washed with TBST again. Ultimately, the protein bands were developed with an enhanced chemiluminescence substrate reaction kit (Thermo Fisher Scientific, Waltham, MA, United States). GAPDH was employed as the internal control.

### Dual-Luciferase Reporter Assay

The binding sequence between ORC6 and miR-1-3p was co-predicted with miRmap, microT, and miRanda. Accordingly, wild type ORC6 3′UTR sequence (ORC6 WT) and mutant type ORC6 3′UTR sequence (ORC6 Mut) were sub-cloned into pmirGLO dual-luciferase miRNA target expression vector (Promega Corp., Madison, WI, United States), and then, respectively, transfected into Huh7 cells, together with miR-1-3p mimics or control miRNAs. Thirty-six hours later, the luciferase activity was examined by a dual-luciferase reporter gene analysis system (Promega, Madison, WI, United States).

### Statistical Analysis

All of the data were processed utilizing SPSS 23.0 software (SPSS Inc., Chicago, IL, United States). Data were represented as mean ± standard deviation (SD). The Student’s *t-*test was employed for analyzing the differences between the two groups. One-way ANOVA with Tukey’s *post hoc* test was utilized for analyzing the differences among multiple groups. *P* < 0.05 indicated statistical significance.

## Results

### Expression of miR-1-3p Was Downregulated in HCC

**FIGURE 1:**
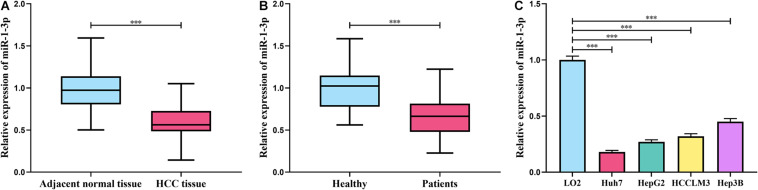
MiR-1-3p was downregulated in HCC. **(A)** Expression of miR-1-3p in HCC tissues and adjacent tissues was detected using qRT-PCR. **(B)** Expression of miR-1-3p in the serum of HCC patients and healthy controls was detected using qRT-PCR. **(C)** Expression of miR-1-3p in HCC cell lines and normal liver cells was examined by qRT-PCR. *** represents *p* < 0.001, respectively.

We firstly analyzed the HCC miRNA expression profile data from the Gene Expression Omnibus (GEO) database^1^ (GEO accession no: GSE108724), and it indicated that miR-1-3p expression was markedly reduced in HCC samples compared with adjacent normal tissues (Supplementary Figure 1A). Additionally, ENCORI database^2^ also indicated that miR-1-3p was downregulated in liver cancer tissues compared with normal liver tissues (Supplementary Figure 1B). Besides, importantly, the K-M plotter database^3^ suggested that HCC patients with high miR-1-3p expression had a favorable prognosis (Supplementary Figure 1C). Subsequently, the expressions of miR-1-3p in HCC tissues, HCC patients’ serum, and HCC cell lines were examined by qRT-PCR. As expected, miR-1-3p expression in HCC tissue was downregulated compared with the adjacent liver tissues, and it was downregulated in HCC patients’ serum (Figures 1A,B); consistently, miR-1-3p expression was downregulated in HCC cell lines by comparison with normal liver cell L02 (Figure 1C). Collectively, these data implied that miR-1-3p may function as a tumor suppressor in HCC.

### miR-1-3p Suppressed the Malignancy of HCC Cells

**FIGURE 2:**
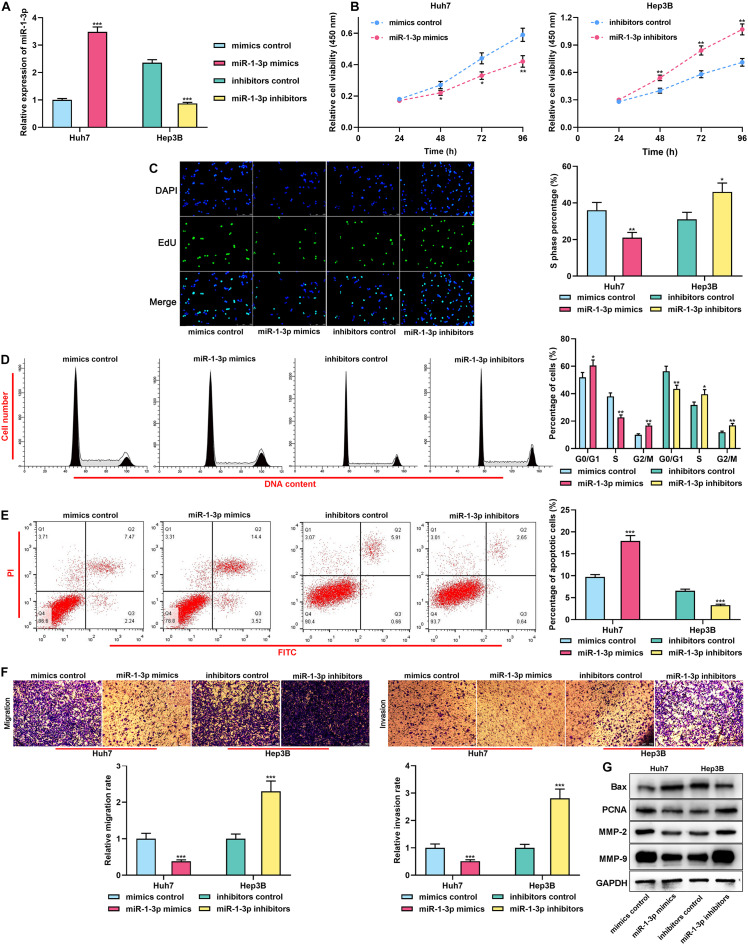
MiR-1-3p inhibited the proliferation, migration, and invasion and promoted apoptosis of HCC cells. **(A)** The transfection efficiency of miR-1-3p mimics and miR-1-3p inhibitors was examined using qRT-PCR. **(B)** After the transfection, the proliferation of Huh7 and Hep3B cells was detected utilizing the CCK-8 assay. **(C)** After the transfection, EdU assay was performed to determine the proliferation of Huh7 and Hep3B cells. **(D)** After the transfection, flow cytometry was performed to analyze the cell cycle distribution of Huh7 and Hep3B cells. **(E)** After the transfection, apoptosis of Huh7 and Hep3B cells was measured using flow cytometry. **(F)** After the transfection, migration and invasion of Huh7 and Hep3B cells were measured through Transwell assay. **(G)** After the transfection, Western blot was used to detect the expressions of Bax, PCNA, MMP-2, and MMP-9 of Huh7 and Hep3B cells. *, **, *** represent *p* < 0.05, *p* < 0.01, and *p* < 0.001, respectively.

For clarifying the biological functions of miR-1-3p in HCC, gain-of-function and loss-of-function models were established. Considering that miR-1-3p expression was the lowest in Huh7 cells and the highest in Hep3B cells, miR-1-3p mimics and miR-1-3p inhibitors were transiently transfected into Huh7 and Hep3B cells, respectively, and the transfection efficiency was confirmed by qRT-PCR (Figure 2A). Functional experiments showed that overexpression of miR-1-3p significantly repressed the proliferation, cell cycle progression, migration, and invasion, but promoted the apoptosis of Huh7 cells, whereas miR-1-3p inhibitors worked oppositely (Figures 2B–F). Western blot indicated that overexpression of miR-1-3p promoted Bax expression and inhibited PCNA, MMP-2, and MMP-9 expression, whereas miR-1-3p inhibitors exerted the opposite effects (Figure 2G). Taken together, it was confirmed that miR-1-3p inhibited the malignant biological behaviors of HCC cells.

### ORC6 Was Confirmed as a Direct Target of miR-1-3p

**FIGURE 3:**
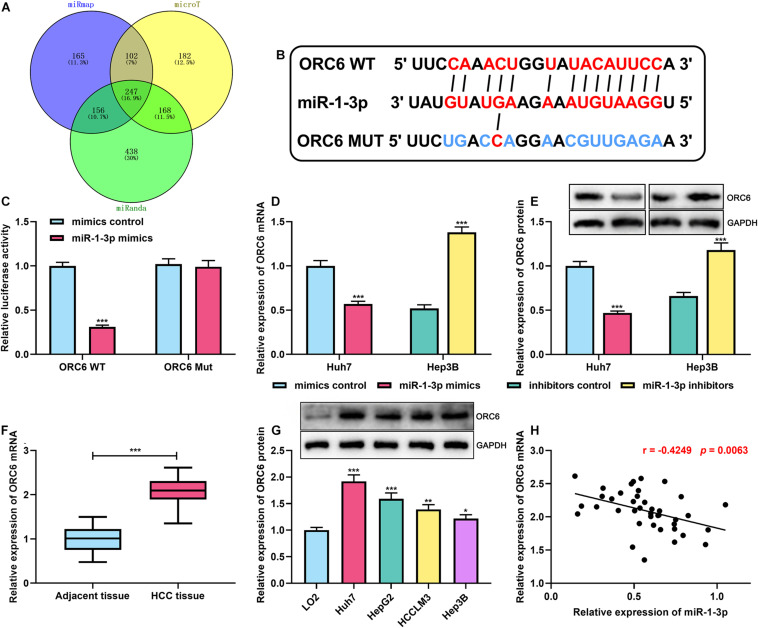
MiR-1-3p directly targeted ORC6. **(A)** Venn diagram showed overlapped target genes of miR–1–3p in miRmap, microT, and miRanda databases. **(B)** The binding site between ORC6 mRNA 3′UTR and miR-1-3p was co-predicted by miRmap, microT, and miRanda databases. **(C)** A dual-luciferase reporter assay was performed to validate the binding site between miR-1-3p and ORC6. **(D)** The effect of miR-1-3p on ORC6 mRNA expression was measured by qRT-PCR. **(E)** The effect of miR-1-3p on ORC6 protein expression was detected by Westen blot. **(F)** Expression of ORC6 in HCC tissues and adjacent liver tissues was examined by qRT-PCR. **(G)** Expression of ORC6 in normal liver cells and HCC cell lines was examined utilizing Western blot. **(H)** The correlation between miR-1-3p expression and ORC6 expression in HCC tissues was analyzed. All of the experiments were performed in triplicate. *, **, and *** represent *p* < 0.05, *p* < 0.01, and *p* < 0.001, respectively.

To further uncover the mechanism of miR-1-3p on HCC progression, through analyzing miRmap,^4^ microT,^5^ and miRanda databases,^6^ we found that there were 247 potential target genes of miR-1-3p, and interestingly, ORC6 was among them (Figure 3A). Afterward, GO and KEGG analyses of these 247 genes were performed using the DAVID database.^7^ GO analysis unveiled that some biological processes were enriched, including “cell division,” “mitotic nuclear division,” and “cell proliferation” (Supplementary Figure 1D). KEGG analyses indicated that the genes were most enriched in the cell cycle pathway (Supplementary Figure 1E). ORC6 is a crucial regulator in cell cycle progression (Chesnokov et al., 2003). Both GEPIA database and ENCORI database suggested that ORC6 expression was remarkably upregulated in HCC tissues compared with normal liver tissues (Supplementary Figures 1F,G). Additionally, survival analysis was further performed using the data of GEPIA and ENCORI databases, the samples were stratified into high and low ORC6 expression groups based on the median value of ORC6 expression, and it was demonstrated that high expression of ORC6 was associated with a detrimental prognosis of HCC patients (Supplementary Figures 1H,I). Additionally, the data from the LinkedOmics database^8^ showed that high ORC6 expression was linked to lymph node metastasis, advanced clinical stage, and high tumor grade (Supplementary Figures 1J–L). What is more, the reanalysis of the HCC gene expression profile data from the GEO database (GEO accession no: GSE84402 and GSE101685) indicated that ORC6 expression was markedly upregulated in HCC tissues, especially the HCC tissues in T1 and T3 stages compared with normal liver tissues (Supplementary Figures 2A–D). Thus, we supposed that ORC6 might be involved in the regulation of HCC progression.

For verifying our hypothesis that ORC6 was a direct target of miR-1-3p, the binding site between ORC6 mRNA 3′UTR and miR-1-3p was co-predicted utilizing miRmap, microT, and miRanda (Figure 3B). Dual-luciferase reporter assay was conducted in Huh7 cells, the findings of which suggested that transfection of miR-1-3p mimics remarkably repressed the luciferase activity of the ORC6 WT reporter; however, when the binding site was mutated, miR-1-3p could not inhibit the luciferase activity of the reporter (Figure 3C). As expected, it was observed that miR-1-3p overexpression repressed ORC6 expression at both mRNA and protein levels, whereas miR-1-3p inhibitors promoted ORC6 expression (Figures 3D,E). What is more, qRT-PCR indicated that ORC6 expression in HCC tissues was significantly upregulated in HCC samples (Figure 3F). Similarly, Western blot showed that compared with normal liver cell L02, ORC6 expression in HCC cell lines was markedly increased (Figure 3G). Besides, the expression of ORC6 mRNA was in a negative correlation with miR-1-3p in HCC tissues (Figure 3H). These results validated that ORC6 was a target gene of miR-1-3p and could be negatively regulated by miR-1-3p.

### ORC6 Promoted the Malignant Biological Behaviors of HCC Cells

**FIGURE 4:**
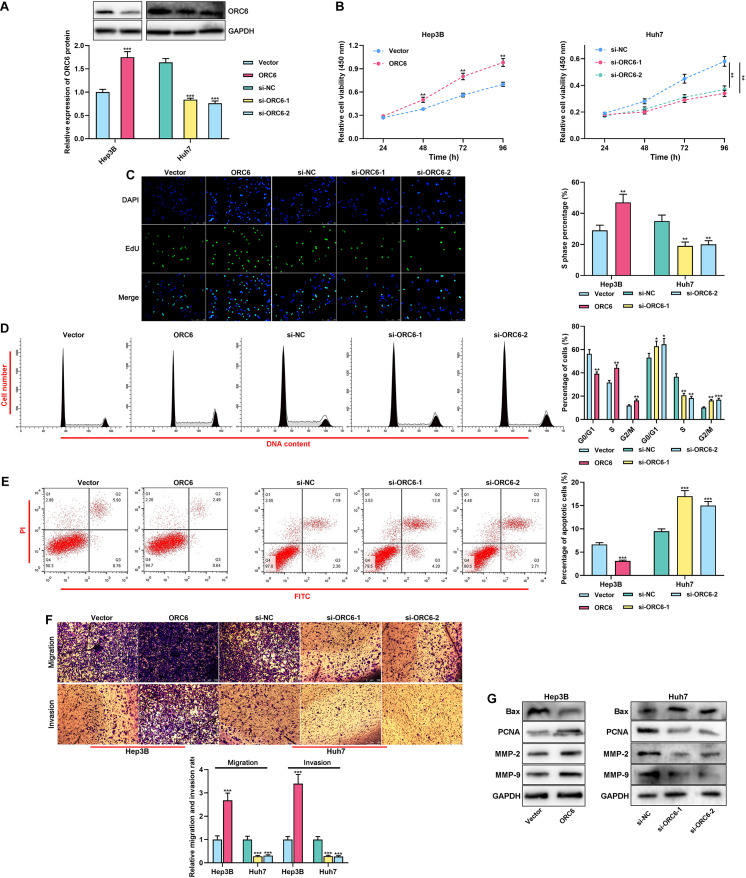
ORC6 regulates the malignant biological behaviors of HCC cells. **(A)** Transfection efficiency of ORC6 overexpression plasmids and siRNAs was examined using Western blot. **(B)** After the transfection, the proliferation of Huh7 and Hep3B cells was detected employing a CCK-8 assay. **(C)** After the transfection, EdU assay was performed to determine the percentage of cells in the S phase. **(D)** After the transfection, flow cytometry was performed to analyze the cell cycle distribution of Huh7 and Hep3B cells. **(E)** After the transfection, apoptosis of Huh7 and Hep3B cells was validated using flow cytometry. **(F)** After the transfection, migration and invasion ability of Huh7 and Hep3B cells were measured through Transwell assay. **(G)** After the transfection, Western blot was used to detect the expressions of Bax, PCNA, MMP-2, and MMP-9 of Huh7 and Hep3B cells. All of the experiments were performed in triplicate. *, **, and *** represent *p* < 0.05, *p* < 0.01, and *p* < 0.001, respectively.

For verifying whether ORC6 could promote the malignant phenotypes of HCC cells, ORC6 overexpression plasmids and the empty plasmids were, respectively, transiently transfected into Hep3B cells, and si-ORC6-1, si-ORC6-2, and the si-NC were, respectively, transiently transfected into Huh7 cells. Western blot was performed to validate the transfection efficiency (Figure 4A). As shown, ORC6 overexpression remarkably promoted the proliferation, cell cycle progression, migration, and invasion, but repressed the apoptosis of HCC cells, while ORC6 depletion showed opposite effects (Figures 4B–F). Western blot results indicated that overexpression of ORC6 inhibited Bax expression and promoted PCNA, MMP-2, and MMP-9 expression, whereas ORC6 knockdown exerted the opposite effects (Figure 4G). These data implied that ORC6 promoted the malignant biological behaviors of HCC cells.

**FIGURE 5:**
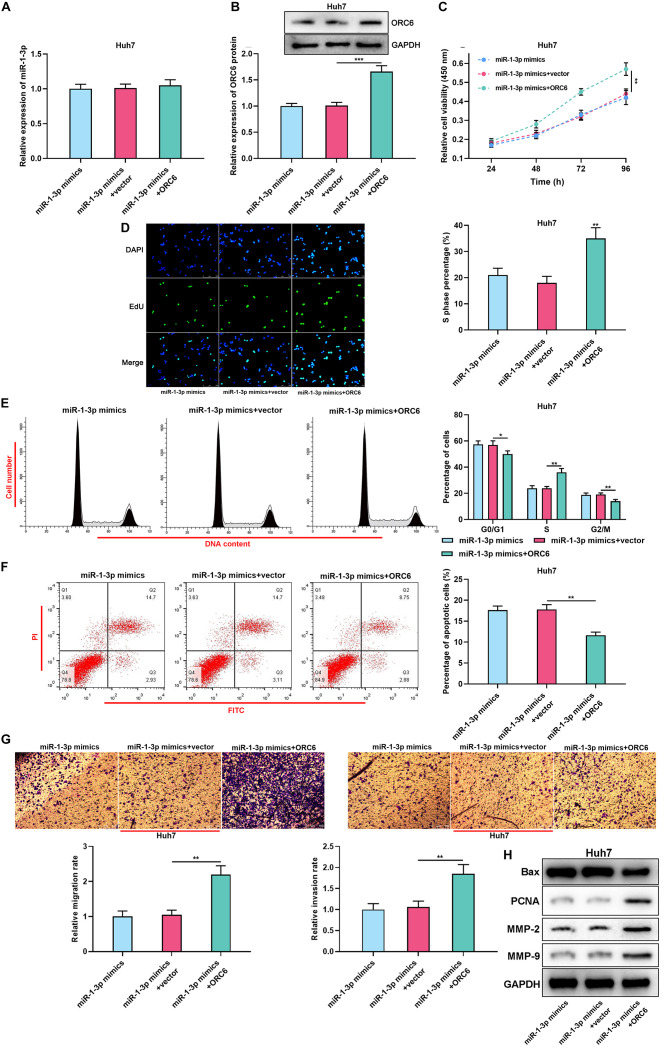
MiR-1-3p/ORC6 axis regulates the malignant biological behaviors of Huh7 cells. Huh7 cells were transfected with miR-1-3p mimics, or miR-1-3p mimics + empty vector, or miR-1-3p mimics + ORC6 overexpression plasmids, respectively. **(A)** The relative expression of miR-1-3p was detected with qRT-PCR. **(B)** The relative protein expression level of ORC6 was measured using Western blot. **(C)** CCK-8 assay was conducted to detect Huh7 cell proliferation. **(D)** EdU assay was performed to determine the percentage of cells in the S phase. **(E)** Flow cytometry was performed to analyze Huh7 cell cycle distribution. **(F)** Apoptosis of Huh7 cells was examined by flow cytometry. **(G)** Huh7 cell migration and invasion were measured using Transwell assay. **(H)** Western blot was used to detect the expression of Bax, PCNA, MMP-2, and MMP-9 of Huh7 and Hep3B cells. All of the experiments were performed in triplicate. *, **, and *** represent *p* < 0.05, *p* < 0.01, and *p* < 0.001, respectively.

### Overexpression of ORC6 Abrogated the Effects of miR-1-3p

For further investigating the role of the miR-1-3p/ORC6 axis in HCC progression, ORC6 overexpression plasmid and miR-1-3p mimics were co-transfected into Huh7 cells. Then, qRT-PCR and Western blot were conducted to detect the expression of miR-1-3p and ORC6 after transfection (Figures 5A,B). Then, “rescue experiments” were performed. As expected, ORC6 overexpression reversed the inhibitory effect of miR-1-3p overexpression on Huh7 cell proliferation, cell cycle progression, migration, invasion, PCNA, MMP-2, and MMP-9 expression, and weakened the promotion of miR-1-3p overexpression on Huh7 cell apoptosis and Bax expression (Figures 5C–H). Collectively, we concluded that miR-1-3p repressed the proliferation, migration, and invasion and promoted the apoptosis of HCC cells by targeting ORC6.

## Discussion

In recent years, the relationship between miR-1-3p and human diseases has been gradually unveiled. MiR-1-3p is reported to participate in the pathogenesis of cardiovascular diseases, endocrine diseases, and cancers (Gerlinger-Romero et al., 2017; Jiao et al., 2018; Li M. et al., 2018; Ke et al., 2019; Zhang et al., 2019). In cancer biology, miR-1-3p has been identified as a tumor suppressor in multiple types of cancers. In lung cancer, hepatocyte growth factor induces gefitinib resistance *via* repressing miR-1-3p expression in PC-9 and HCC827 cells, whereas miR-1-3p restoration abolishes the drug resistance by inhibiting c-Met signaling (Jiao et al., 2018). MiR-1-3p expression is reported to be downregulated in gastric cancer tissues and cell is associated with tumor size, and transfection of miR-1-3p notably impedes the proliferation and invasion of MGC-803 cells (Ke et al., 2019). In HCC, reportedly, miR-1-3p represses the proliferation of cancer cells through targeting SOX9 (Zhang et al., 2019). In the present study, by performing gain-of-function and loss-of-function experiments with Huh7 and Hep3B cells, we demonstrated that miR-1-3p induced cell cycle arrest, impeded proliferation, migration, and invasion, and induced apoptosis of HCC cells, which is consistent with the previous report (Zhang et al., 2019). These findings suggest that miR-1-3p may be a promising target for HCC treatment.

Interestingly, in human diseases, miR-1-3p shows the potential as a biomarker. Reportedly, upregulation of miR-1-3p in peripheral blood is observed in patients with gestational hypertension (GH) or preeclampsia (PE) and miR-1-3p (Hromadnikova et al., 2019a). Similarly, miR-1-3p is also aberrantly expressed in the peripheral blood of children descending from pregnancies with GH or PE (Hromadnikova et al., 2019b). Interestingly, in the present work, it was demonstrated that miR-1-3p was upregulated in both the HCC tissues and serum samples of the patients, and the expression level of miR-1-3p in HCC tissues was associated with the prognosis of the patients. These data suggest that miR-1-3p may be a promising biomarker for the diagnosis and prognosis prediction of HCC. However, this should be validated with a larger number of patients from different medical centers in the following work.

The current study verified that ORC6 was directly regulated by miR-1-3p, and ORC6 counteracted the biological effects of miR-1-3p on HCC cells. The origin recognition complex (ORC), a conserved complex consisting of six subunits, is essential for the initiation of the DNA replication, serving as the platform for the assembly of other initiation factors such as Cdc6 and Mcm proteins (Li N. et al., 2018). ORC6 is the smallest ORC subunit. ORC6 partakes in the positioning of ORC at the origins of DNA replication. The middle domain of human ORC6 has an overall fold similar to the corresponding helical domain of transcription factor TFIIB, and several amino acid residues of ORC6 are identified to contribute to DNA binding and recognition (Prasanth et al., 2002; Liu et al., 2011). Reportedly, upregulated ORC6 expression is indicative of a poor clinical outcome in colorectal cancer patients (Chesnokov et al., 2003). Another research reports that depletion of ORC6 increases the sensitivity of colonic cancer cell line HCT116 to cisplatin (Gavin et al., 2008). In the present work, we demonstrated that ORC6 was significantly upregulated in HCC tissues; further experiments showed that ORC6 overexpression markedly facilitated the proliferation, cell cycle, migration, and invasion of HCC cells but repressed apoptosis. Our data suggest that ORC6 is a novel tumor-promoting factor in HCC, and the downregulation of miR-1-3p contributes to its dysregulation in HCC. However, the downstream mechanism by which ORC6 participates in HCC progression remains unclear, which requires further investigation in the future.

All in all, in the present work, we report the expression, biological functions, and regulatory mechanism of the tumor suppressor miR-1-3p and the tumor-promoting factor ORC6 in HCC. Our findings suggest that miR-1-3p/ORC6 axis participates in HCC progression. This work provides novel clues for the diagnosis and therapy of HCC. In the following studies, animal models are required to further validate the role of miR-1-3p/ORC6 axis in HCC progression.

## Data Availability Statement

The original contributions presented in the study are included in the article/Supplementary Material, further inquiries can be directed to the corresponding author/s.

## Ethics Statement

This work is supported by the Ethics Committee of Hubei Cancer Hospital, and written informed consents of the patients were obtained. The patients/participants provided their written informed consent to participate in this study.

## Author Contributions

XT conceived and designed the experiments. HC and LB performed the experiments. HC and DW performed the statistical analysis. All the authors took part in the manuscript writing. All authors read and approved the final manuscript.

## Conflict of Interest

The authors declare that the research was conducted in the absence of any commercial or financial relationships that could be construed as a potential conflict of interest.

## Publisher’s Note

All claims expressed in this article are solely those of the authors and do not necessarily represent those of their affiliated organizations, or those of the publisher, the editors and the reviewers. Any product that may be evaluated in this article, or claim that may be made by its manufacturer, is not guaranteed or endorsed by the publisher.

## Footnotes

1
https://www.ncbi.nlm.nih.gov/

2
http://starbase.sysu.edu.cn/

3
http://kmplot.com/analysis/index.php

4
https://mirmap.ezlab.org/

5
http://diana.imis.athena-innovation.gr/

6
http://www.miranda.org/

7
https://david.ncifcrf.gov/

8
http://www.linkedomics.org/login.php
