# Supplementary material for: ORC6, Negatively Regulated by miR-1-3p, Promotes Proliferation, Migration, and Invasion of Hepatocellular Carcinoma Cells
Source: Front Cell Dev Biol. 2021 Jul 29;9:652292. doi: 10.3389/fcell.2021.652292 (PMC8358308; doi:10.3389/fcell.2021.652292)
Supplement: Supplementary file 1 [file Data_Sheet_1.docx]

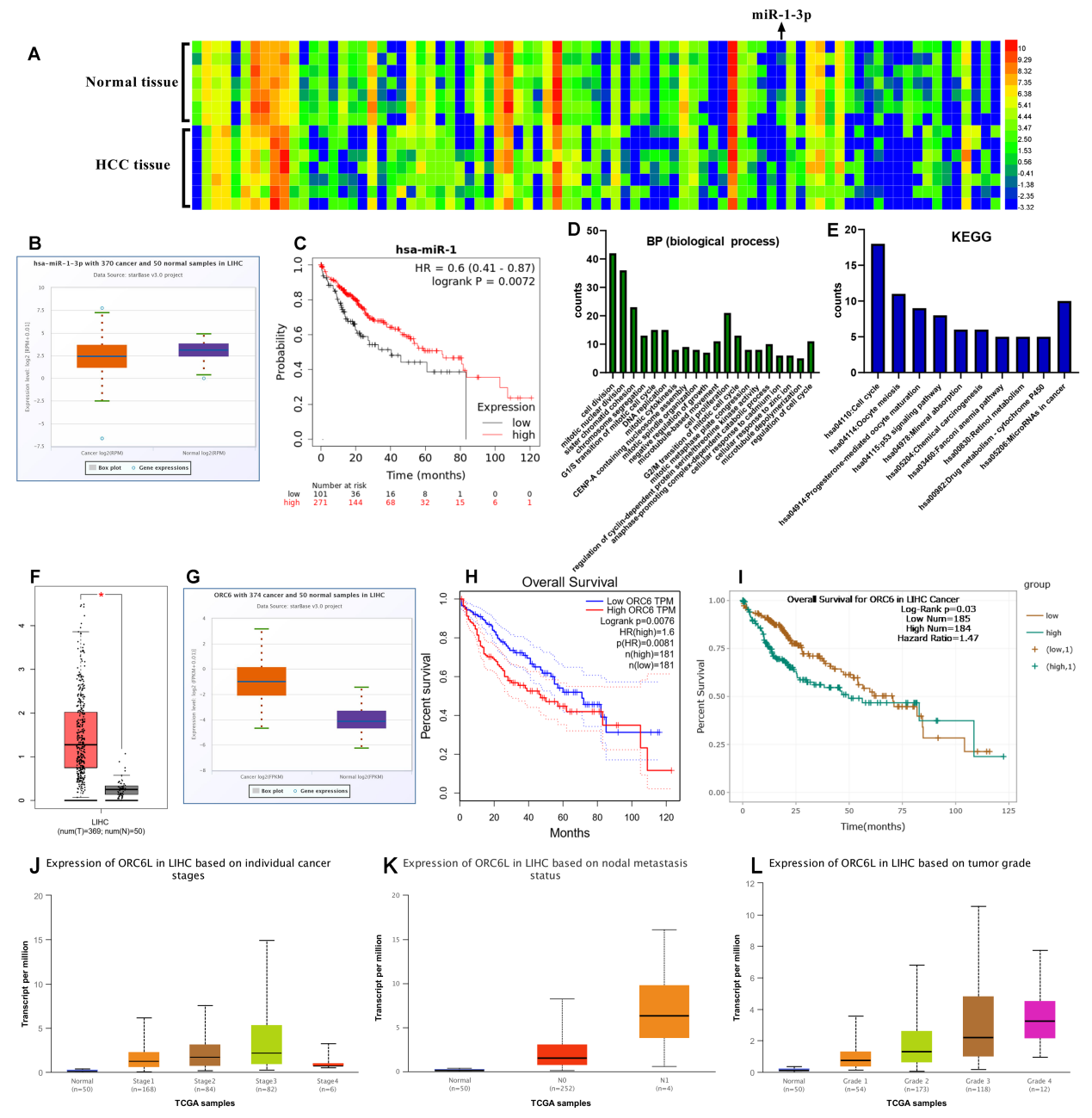


**Supplementary Figure 1 Bioinformatics analysis suggested that miR-1-3p and ORC6 were associated with HCC progression.**

(A) In dataset GSE108724, expression profile of miRNAs in HCC tissues and normal liver tissues was shown using heatmap.

(B) MiR-1-3p expression in HCC tissues (Orange column) and normal tissues (purple column) were analyzed by ENCORI database.

(C) K-M plotter database was used to analyze the survival time of HCC patients with high miR-1-3p expression or low miR-1-3p expression.

(D, E) These overlapped target genes of miR-1-3p were processed with GO enrichment and KEGG pathway analysis.

(F, G) Expression of ORC6 in HCC tissues and normal liver tissues was analyzed by GEPIA and ENCORI database.

(H, I) Survival analysis of HCC patients based on ORC6 expression level was performed by GEPIA and ENCORI.

(J-L) The relationship between ORC6 expression and nodal metastasis, individual cancer stage and tumor grade was analyzed by LinkedOmics database.


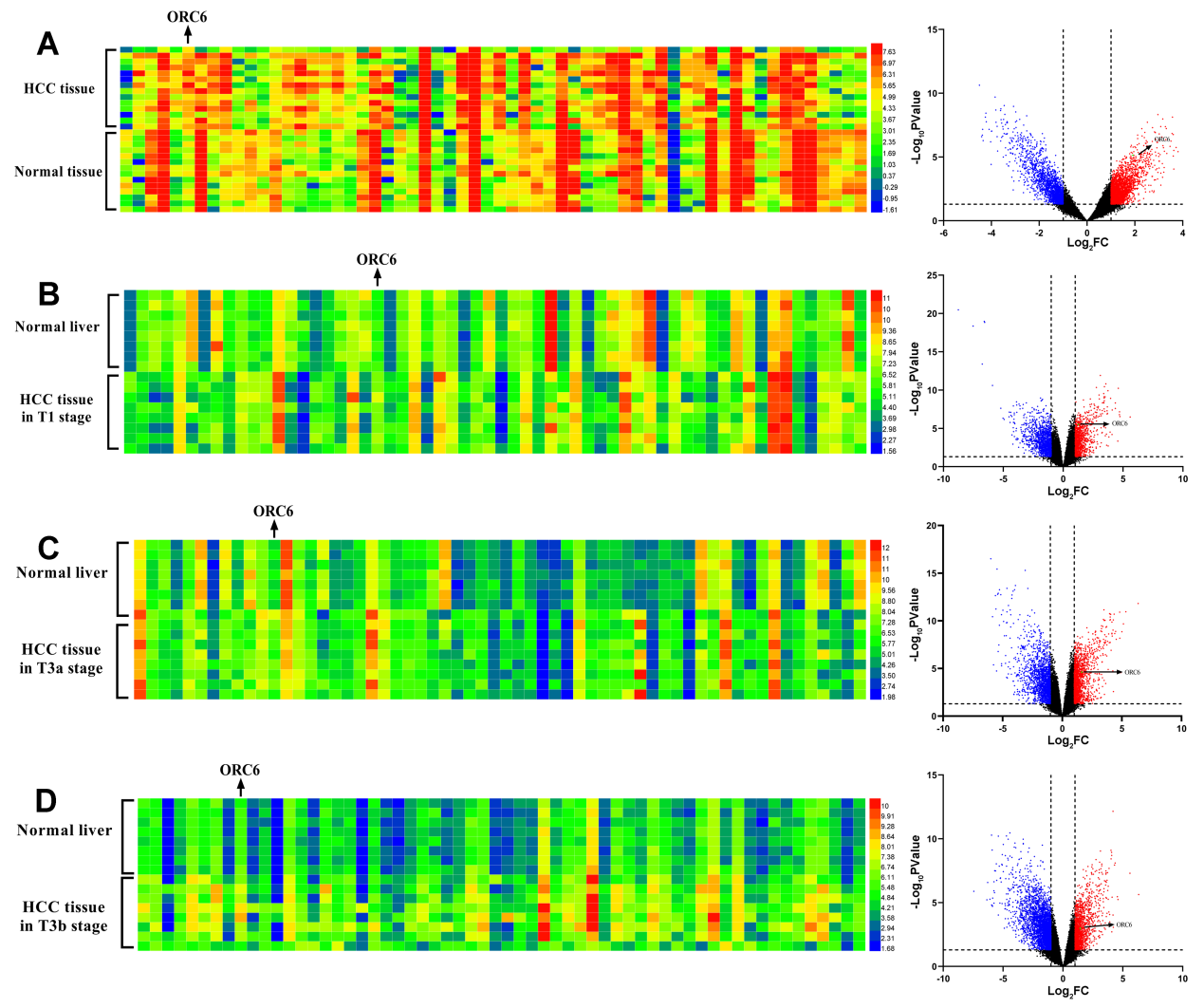


**Supplementary Figure 2 Bioinformatics analysis suggested ORC6 was up-regulated in HCC.**

1. In dataset GSE84402, expression profile of genes in HCC tissues and normal liver tissues was shown using heatmap and volcano plot.

(B-D) In dataset GSE101685, expression profile of genes in normal liver tissues and HCC tissues (clinical stages TI, T3a and T3b) was shown through heatmap and volcano plot.

In volcano plots, significantly upregulated (*p* < 0.05 and log_2_FC > 1) and downregulated genes (*p* < 0.05 and log_2_FC < -1) were marked in red and blue, respectively.
